# Neoadjuvant chemotherapy with angiogenesis inhibitor in early-stage breast cancer: a systematic review and meta-analysis

**DOI:** 10.3389/fonc.2026.1830676

**Published:** 2026-07-15

**Authors:** Jiangzhuo Wu, Hanbing Li, Ling Wei, Xiao Yan, Jiang Fang, Lin Peng, Xiaobo Zhao

**Affiliations:** 1 Department of Thyroid and Breast Surgery, Affiliated Hospital of North Sichuan Medical College, Nanchong, Sichuan, China; 2 National Clinical Key Specialty (General Surgery)/National Clinical Research Center for Digestive Diseases/Sichuan Clinical Research Center for Digestive Diseases, Nanchong, Sichuan, China

**Keywords:** angiogenesis inhibitor, breast cancer, meta-analysis, neoadjuvant chemotherapy, survival analysis

## Abstract

**Background:**

Breast cancer, as a prevalent malignant tumor among women worldwide, has its malignant progression closely linked to angiogenesis mechanisms. Research indicates that combining anti-angiogenic drugs can promote tumor vascular normalization, thereby improving the delivery of chemotherapeutic agents and reversing the drug-resistant microenvironment. Some clinical studies have confirmed that such combination regimens can enhance pathological response rates (pCR). This paper aims to systematically evaluate the clinical value of angiogenesis inhibitors in neoadjuvant chemotherapy (NACT) for breast cancer and provide evidence-based guidance to optimize treatment strategies.

**Methods:**

We systematically searched the PubMed and Web of Science databases up to July 16, 2025, to identify randomized controlled trials (RCTs) comparing angiogenesis inhibitor-based NACT regimens with angiogenesis inhibitor-free regimens in breast cancer patients. Using random effects models, we calculated pooled odds ratios and hazard ratios with 95% confidence intervals for pCR, disease-free survival (DFS), overall survival (OS), objective response rate (ORR), and adverse events (AEs).

**Results:**

This analysis included 10 RCTs (8,069 patients). The results showed that NACT containing angiogenesis inhibitors significantly increased the pCR rate (22.59% vs. 29.88%). Subgroup analysis indicated no statistically significant improvement in pCR among hormone receptor-positive patients, whereas in triple-negative breast cancer (TNBC), the pCR rate was significantly elevated (32.42% vs. 42.46%). Regarding survival outcomes, no significant differences were observed in either DFS or OS.

**Conclusions:**

The combination of bevacizumab with NACT can improve the pCR rate, with this effect being particularly pronounced in TNBC. However, current research has not yet reached a consensus on whether it can improve OS, and there remains a lack of clear biomarkers for predicting treatment efficacy.

**Systematic review registration:**

https://www.crd.york.ac.uk/PROSPERO/, identifier CRD420261411787.

## Introduction

Breast cancer, as one of the most common malignant tumors among women worldwide and a leading cause of cancer-related mortality, is not a single disease entity but rather a heterogeneous collection of subtypes with distinct molecular characteristics, clinical behaviors, and treatment responses. Its malignant progression exhibits high heterogeneity and invasiveness, a process closely linked to angiogenesis mechanisms. Tumor vasculature often displays abnormal features such as tortuosity and leakage, resulting in disordered blood flow, tissue hypoxia, and uneven drug distribution (1). This pathological vascular microenvironment not only supports tumor growth by supplying oxygen and nutrients but also serves as a critical driver of metastasis and a prognostic indicator for poor outcomes (2). Tumor neovascularization, a classic hallmark of cancer, plays a central regulatory role in tumor development and progression, relying on a dynamic balance between pro-angiogenic and anti-angiogenic signals (3). When this balance is disrupted, triggering the activation of the “angiogenic switch,” tumors surpass the 1–3 mm growth limitation and enter a phase of rapid proliferation and metastasis. The hypoxic conditions and drug delivery barriers caused by abnormal vasculature further undermine the efficacy of conventional therapies, creating a vicious cycle (4).

NACT serves as a pivotal treatment strategy for early-stage breast cancer patients and has become a cornerstone of comprehensive breast cancer management. Its therapeutic objectives have evolved from initially shrinking tumors to facilitate surgical procedures (such as breast-conserving surgery) to serving as a critical platform for assessing *in vivo* chemosensitivity of tumors. By evaluating tumor responses during NACT, clinicians can obtain essential insights to guide subsequent adjuvant treatment strategies (5). Despite its established efficacy, the clinical application of NACT faces multiple challenges: 1. Aberrant tumor vasculature creates hypoxic microenvironments and results in uneven drug distribution, reducing effective chemotherapeutic concentrations in tumor tissues (6); 2. Tumors can evade chemotherapy-induced cytotoxicity through compensatory molecular mechanisms, such as angiogenesis-related signaling pathways, where VEGF/VEGFR2 axis inhibition frequently leads to rapid drug resistance (7); 3. NACT agents (e.g., anthracyclines and taxanes) may induce long-term vascular endothelial dysfunction, increasing the risk of cardiovascular diseases (8); 4. Conventional anti-angiogenic agents (e.g., monoclonal antibodies) exhibit limited efficacy due to poor tumor penetration, while tyrosine kinase inhibitors (TKIs) are associated with off-target effects (9); 5. Overly potent angiogenesis inhibition may exacerbate hypoxia, paradoxically promoting drug resistance and tumor recurrence (6). These limitations underscore the necessity for optimizing treatment strategies, such as combining anti-angiogenic agents to promote tumor vascular normalization, thereby enhancing chemotherapeutic drug delivery and therapeutic efficacy.

Numerous clinical trials have demonstrated that the combination of anti-angiogenic drugs with NACT exhibits synergistic effects in breast cancer treatment. The core mechanism lies in promoting tumor vascular normalization—specifically, selectively pruning immature tumor vessels and improving the structure and function of the tumor microvasculature, thereby enhancing tissue perfusion efficiency and chemotherapeutic drug delivery (10). This process not only reverses the hypoxic tumor microenvironment and addresses uneven drug distribution but also effectively counteracts breast cancer resistance mechanisms, such as hypoxia-induced drug resistance. For instance, a Phase II trial confirmed that the regimen of anlotinib combined with sintilimab (a PD-1 inhibitor) and chemotherapy significantly improved the pCR rate and ameliorated the tumor microenvironment in patients with stage II–III triple-negative breast cancer (11). Another Phase II trial also indicated that famitinib combined with camrelizumab (an anti-PD-1 monoclonal antibody) and nanoparticle albumin-bound paclitaxel demonstrated clear efficacy and manageable safety in breast cancer treatment (12). Collectively, these studies highlight the therapeutic potential of optimizing chemotherapeutic drug delivery systems and reversing the tumor-resistant microenvironment through vascular normalization strategies.

Current studies indicate that among unselected breast cancer patients, the combination of bevacizumab with standard chemotherapy yields only modest improvements in treatment response rates and progression-free survival (PFS) (13). The primary efficacy outcomes of the BEATRICE trial further revealed no significant difference in invasive DFS between treatment regimens containing bevacizumab and those without (14). Additional research has noted that although chemotherapy combined with bevacizumab can enhance pCR following neoadjuvant therapy, this benefit does not translate into an improvement in OS (15). Based on a rigorous evaluation of existing evidence, this paper aims to systematically elucidate the clinical value of angiogenesis inhibitors in NACT for breast cancer, providing critical insights for academic discussions on optimizing breast cancer treatment strategies. This systematic review will conduct an in-depth analysis of the clinical benefits and potential risks associated with angiogenesis inhibitor use, focusing on the controversial issues surrounding their application in neoadjuvant therapy, with the goal of offering evidence-based guidance for clinical practice.

## Methods

Ethics approval is not necessary. This is a meta-analysis; this study only collected data that had already been publicly published.

This systematic review and meta-analysis synthesizes quantitative evidence from randomized controlled trials to evaluate the efficacy and safety of angiogenesis inhibitors (experimental group) versus no angiogenesis inhibitors (control group) in breast cancer patients. The methodology was meticulously designed to ensure the robustness and reliability of the study findings.

## Search strategy and study identification

A comprehensive literature search was conducted in the PubMed and Web of Science databases without language or date restrictions, concluding on July 16, 2025. The search strategy incorporated a combination of specific keywords and free-text terms using Boolean operators to identify relevant studies. Keywords included “breast cancer”, “Angiogenesis Inhibitor”, “Bevacizumab”, “Sunitinib”, “Sorafenib”, and “Anlotinib”. A detailed search expression is provided in the **Supplementary Materials**. In addition to the database searches, cross-referencing of relevant articles was employed to capture all potentially pertinent records. We acknowledge that searching only these two databases may have limitations in terms of incomplete literature coverage. Two independent reviewers (Wu JZ and Y X) executed the systematic literature search, with discrepancies resolved through consultation with a third reviewer (P L).

## Selection criteria and data extraction

### Selection criteria and data extraction

For eligibility to be included in the meta-analysis, the study must meet the following criteria: Phase II or III randomized controlled trials, including NACT for breast cancer patients receiving angiogenesis inhibitors in the experimental group and patients receiving no angiogenesis inhibitors in the control group. Only two groups of pCR rates were included in the study to estimate OR and 95% CI. If the study is a non-randomized controlled trial that has studied the angiogenic inhibitor NACT in cancer subtypes other than breast cancer, or is in progress, but cannot obtain results during literature retrieval, it will be excluded. The following variables were extracted from each included RCT: study name, population characteristics, intervention details, and results related to pCR rate.

### Study objectives

The main concern is the absence of residual invasive tumors in the breast or axilla after treatment. This analysis includes four main categories: (1) all RCTs, regardless of chemotherapy backbone and molecular typing. (2) RCT of HR (+) patients. (3) RCT of TNBC patients.

Secondary objectives included evaluating the activity, efficacy, and safety of angiogenic inhibitor NACT in breast cancer patients, including ORR, OS, DFS, stable disease (SD), progressive disease (PD), and adverse events such as neutropenia, hypertension, febrile neutropenia, mucositis, and nausea.

### Bias risk assessment

We have conducted a formal bias risk assessment of the 10 randomized controlled trials included using the Cochrane RoB 2.0 tool (**Table 1**). The evaluation covers five areas: randomization process, deviation intervention, missing outcome data, outcome measurement, and selective reporting. The evaluation results showed that the overall risk of bias for all studies was “Some concerns” (mainly due to open label design).

**Table 1:** Bias risk assessment.

Study	Randomization process	Deviation intervention	Missing outcome data	Outcome measurement	Selective reporting	Overall risk of bias
NCT00408408	Low	Some concerns	Low	Low	Low	Some concerns
NCT00528567	Low	Some concerns	Low	Low	Low	Some concerns
NCT00567554	Low	Some concerns	Low	Low	Low	Some concerns
NCT00773695	Low	Some concerns	Low	Low	Low	Some concerns
NCT00856492	Low	Some concerns	Some concerns	Low	Low	Some concerns
NCT00861705	Low	Some concerns	Low	Low	Low	Some concerns
NCT01093235	Low	Some concerns	Low	Low	Low	Some concerns
NCT01142778	Low	Some concerns	Some concerns	Low	Low	Some concerns
NCT01190345	Low	Some concerns	Some concerns	Low	Low	Some concerns
PMID: 24136883	Low	Some concerns	Low	Low	Low	Some concerns

### Statistical analysis

The OR and 95% CI of the effects of adding the angiogenesis inhibitor NACT on pCR, ORR, and AE were calculated. An OR greater than 1 indicates a higher incidence of pCR, ORR, and AE in the NACT group, while an OR less than 1 indicates a lower incidence in this group. We used the Mantel-Haenszel method to obtain a fixed effects model with merged OR and conducted a standard test on the homogeneity hypothesis (16). In the presence of significant heterogeneity between experiments, the combined estimates of OR were calculated using the DerSimonian and Laird methods through a random effects model. Calculate the Higgins I^2^ index to quantify the degree of inconsistency in research results. Evaluate publication bias by visually examining the funnel plot of study size and treatment efficacy (17) and conducting an asymmetry test using Harbord (18). If the 95% confidence interval does not include 1.0 and the P value is<0.05 (two-sided), it is considered statistically significant. The rule for heterogeneity treatment: If I ^2^<50%, a fixed effects model is used; I ^2^ ≥ 50% adopts a random effects model; When I ^2^ ≥ 75%, do not merge and only provide a descriptive summary. Sensitivity analysis aims to determine the stability of the merged OR estimates by recalculating the merged OR estimates after excluding each individual study. All statistical analyses and forest land generation were conducted using RevMan software version 5.4.

## Results

**Figure 1:**
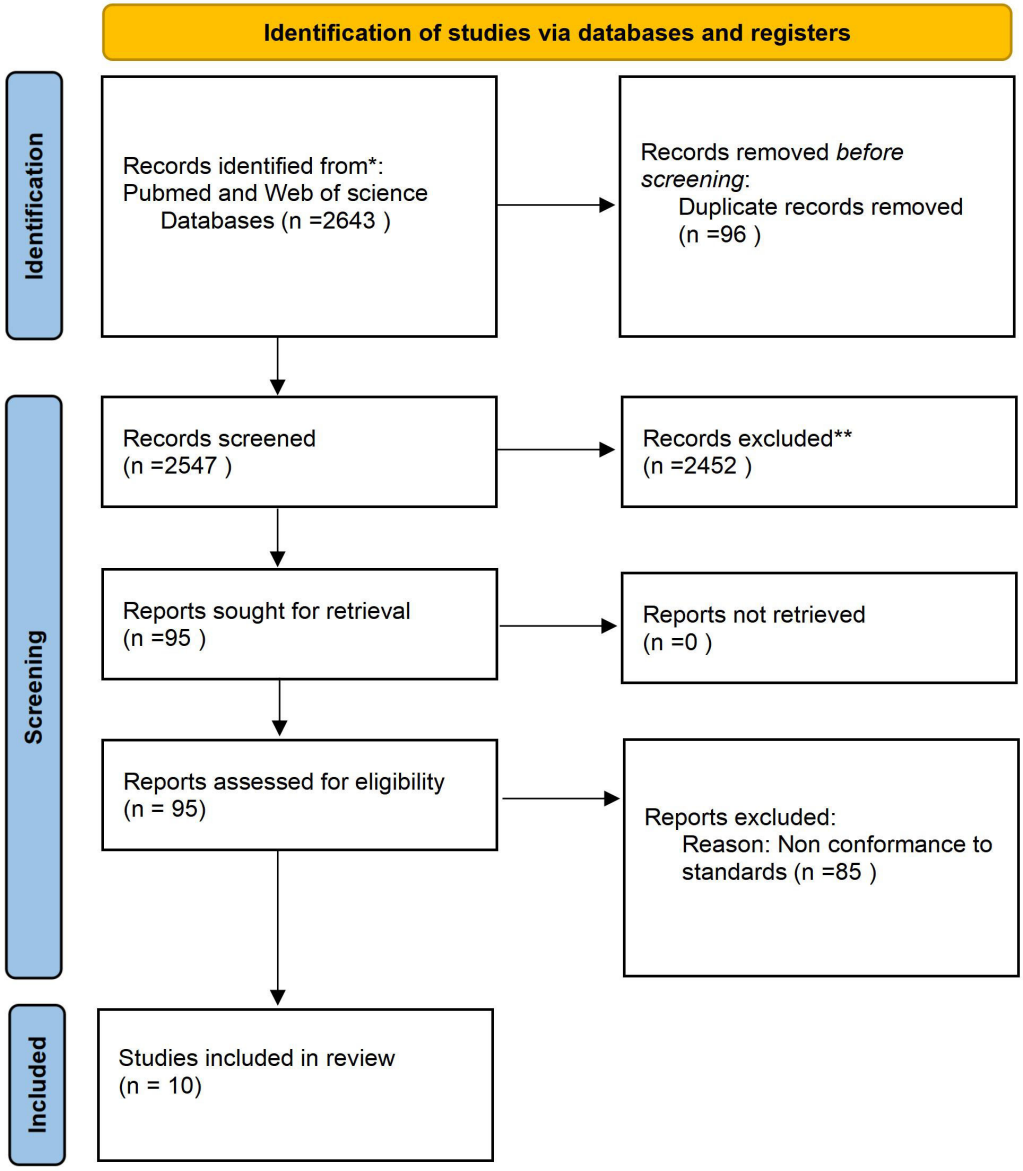
PRISMA 2020 flow diagram for new systematic reviews which included searches of databases and registers only.

After a comprehensive systematic search, 2,537 records were initially identified, and we excluded 2,527irrelevant studies. This left us with 10 potentially eligible RCTs to be included in the final meta-analysis. (**Figure 1**).

This meta-analysis included a total of 8069 breast cancer patients, of whom 4039 (50.05%) received chemotherapy with angiogenesis inhibitors and 4030 (49.95%) received chemotherapy without angiogenesis inhibitors. **Table 2** summarizes the main characteristics of the included RCTs. Among these trials, all 10 RCTs used bevacizumab as the angiogenesis inhibitor, with trastuzumab added for HER-2 positive patients. In 9 randomized controlled trials (n=5,405 patients), both groups received anthracycline- and taxane-based NACT. The same chemotherapy backbone was used in all 10 included randomized controlled trials (n=5,478 patients), regardless of whether angiogenesis inhibitors were administered. Additionally, one RCT (n=226 patients) added carboplatin to the chemotherapy regimen, specifically weekly paclitaxel for 12 weeks, combined with carboplatin every 3 weeks, followed by dose-dense doxorubicin and cyclophosphamide, with or without bevacizumab.

**Table 2:** Main characteristics of the randomized controlled trials included in the present meta-analysis.

Study	Study design	Primary end point	Secondary end points	Treatment arms	Patients, n
NCT00408408 (19)	phase III	ypT0/isN0	ypT0/is pN0, safety, clinical complete responses	T→AC T→AC+Bev TC→AC TC→AC+Bev TG→AC TG→AC+Bev	201 199 204 201 197 204
NCT00528567 (20)	phase III	IDFS	OS, DFS, safety	CT CT+Bev	1290 1301
NCT00567554 (21)	phase III	ypT0/is ypN0	OS, DFS, DDFS, safety	EC-T EC-T+Bev	969 956
NCT00773695 (22)	phase II	ypT0/is ypN0	safety	FEC-T FEC-T+Bev	66 66
NCT00856492 (23)	phase II	ypT0/is ypN0	OS, EFS, safety	nP-AC AC-nP nP+Bev-AC	62 51 98
NCT00861705 (24)	phase II	ypT0/isN0	EFS, OS, safety	wP-ddAC wP-ddAC+Bev wPCarbo-ddAC wPCarbo-ddAC+Bev	115 113 113 113
NCT01093235 (25)	phase III	ypT0/is ypN0	DFS, OS, safety	D-FEC Bev+D-FEC	401 399
NCT01142778 (26)	phase II	ypT0/is ypN0	DFS, OS, DDFS, safety	T+trastuzumab T+trastuzumab+Bev	25 48
NCT01190345 (27)	phase II	ypT0/is ypN0	DFS, OS, safety, RFS	FEC-T FEC-T+Bev	25 50
PMID: 24136883 (28)	phase III	ypT0 ypN0	ypT0/TisypN0;ypT0/TisypN0/+	EC-T EC-T+Bev	340 323

T→AC: four cycles of docetaxel (100 mg per square meter of body-surface area, administered intravenously on day 1 of the cycle) every 3 weeks, followed by four cycles of doxorubicin–cyclophosphamide (60 mg and 600 mg per square meter, respectively, administered intravenously every 3 weeks) (docetaxel group) TC→AC: capecitabine (825 mg per square meter, administered orally twice a day on days 1 through 14) added to docetaxel (75 mg per square meter, administered intravenously on day 1), followed by doxorubicin–cyclophosphamide (docetaxel–cape cit abine group) TG→AC: gemcitabine (1000 mg per square meter, administered intravenously on days 1 and 8) added to docetaxel (75 mg per square meter, administered intravenously on day 1), followed by doxorubicin–cyclophosphamide (docetaxel–gemcitabine group. Bevacizumab (15 mg per kilogram of body weight, administered intravenously, every 3 weeks) with each of the first six cycles of chemotherapy and for 10 additional doses every 3 weeks postoperatively. EC-T: epirubicin (at a dose of 90 mg per square meter of body surface area) plus cyclophosphamide (at a dose of 600 mg per square meter), both administered on day 1, every 3 weeks for four cycles, followed by four cycles of docetaxel at a dose of 100 mg per square meter on day 1, every 3 weeks. Eight cycles of bevacizumab (at a dose of 15 mg per kilogram of body weight intravenously every 3 weeks starting on day 1 of the first epirubicin–cyclophosphamide cycle). FEC-T: 4 cycles of FEC100 (5-fluorouracil 600 mg/m2, epirubicin 100 mg/m2 and cyclophosphamide 600 mg/m2) every 3 weeks, followed by docetaxel 100 mg/m2 every 3 weeks or 12 weekly infusions of paclitaxel 80 mg/m2. Bevacizumab was administered intravenously at a dose of 15 mg/kg every third week or 10 mg/kg every other week in patients receiving docetaxel or paclitaxel, respectively. nP+Bev-AC: (bevacizumab) received intravenous (IV) administration of nab-paclitaxel 100 mg/m2 IV weekly for 12 weeks (nP × 12) with IV bevacizumab 10 mg/kg every 2 weeks (six doses), followed by IV doxorubicin 60 mg/m2 and cyclophosphamide 600 mg/m2 with pegfilgrastim 6 mg subcutaneously every 2 weeks for six cycles (ddAC × 6). nP × 12 followed by ddAC × 6, and those randomized to Arm 3 received ddAC × 6 first followed by nP × 12, both without bevacizumab. Paclitaxel 80 mg/m2 once a week for 12 weeks (wP) and were randomly assigned to the control regimen, with addition of bevacizumab 10 mg/kg once every 2 weeks for 9 doses, carboplatin area under the curve 6 once every 3 weeks for four doses, or both, followed by dose-dense doxorubicin and cyclophosphamide (AC). D-FEC: docetaxel 100 mg/m^2^ once every 21 days for three cycles, followed by fluorouracil 500 mg/m^2^, epirubicin 100 mg/m^2^, with cyclophosphamide 500 mg/m^2^ once every 21 days for three cycles. Bevacizumab 15 mg/kg (Genentech, South San Francisco and Vacaville, CA, USA) was given every 3 weeks with the first four cycles of chemotherapy in the experimental group. T+trastuzumab: two cycles of neoadjuvant docetaxel (100 mg/m^2^) plus trastuzumab (8 mg/kg in cycle 1, and 6 mg/kg thereafter), both administered intravenously (i.v.) every 3 weeks (q3w). Bevacizumab (15 mg/kg i.v. q3w). FEC-T: four 21-day cycles of FEC100 IV infusions (5FU 500 mg/m2, epirubicin 100 mg/m2, and cyclophosphamide 500 mg/m2) on day 1 plus bevacizumab (15 mg/kg on day 1), then received four 21-day cycles of docetaxel (100 mg/m2 on day 1). Bevacizumab (15 mg/kg on day 1). EC-T: epirubicin (E,90mg/m2) plus cyclophosphamide (C,600mg/m2), both administered on day1, every 3 weeks for four cycles, followed by four cycles of docetaxel (D,100mg/m2) on day1, every 3 weeks. Eight cycles of bevacizumab (B,15mg/kgbodyweight) intravenously e very 3 weeks starting on day 1 of the first EC cycle. RFS,relapse-free survival; DFS, disease-free survival; EFS, event-free survival; OS, overall survival; iDFS, invasive DFS.

### Pathological complete response rates analysis

**Figure 2:**
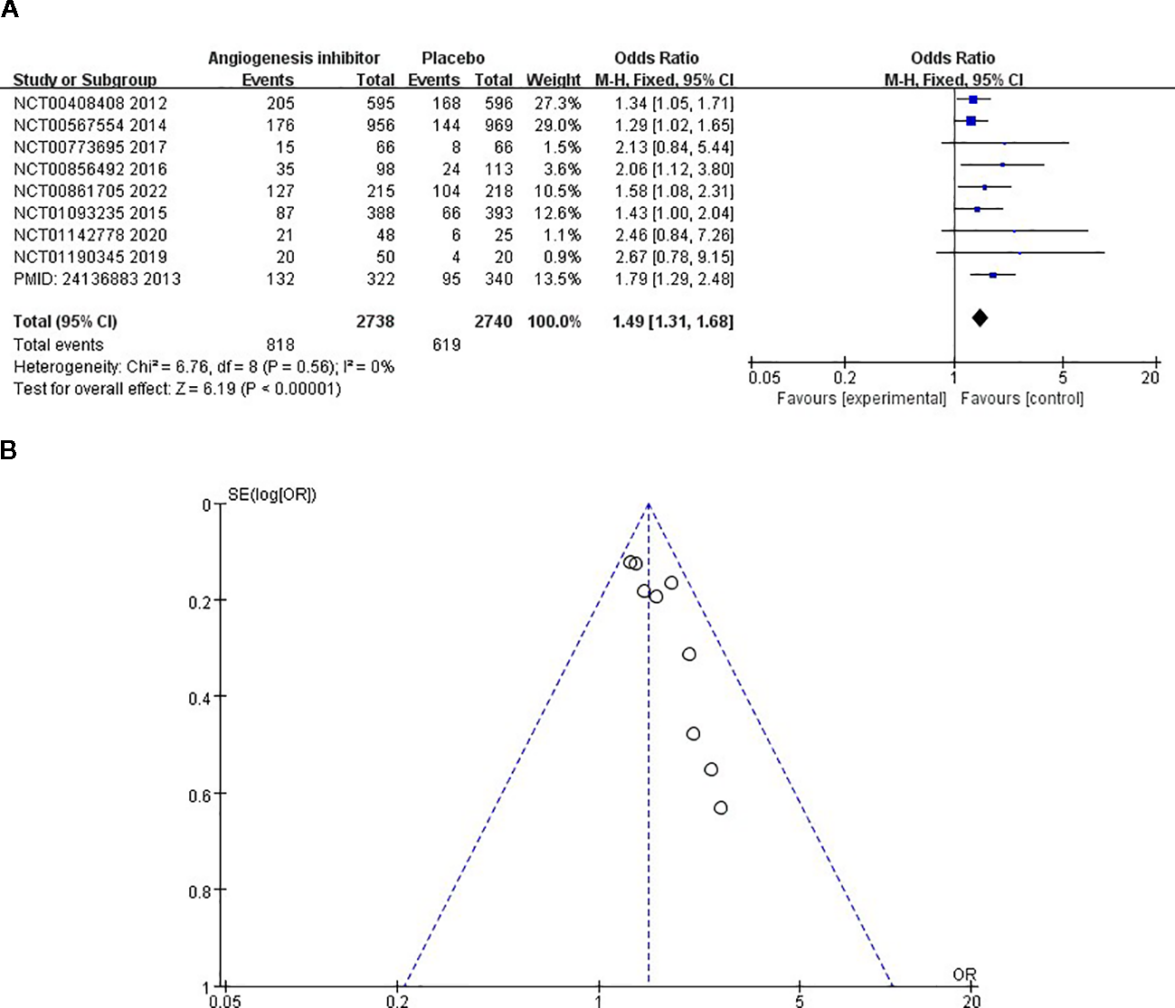
**(A)** Odds ratio for pathological complete response of angiogenesis inhibitor-based versus angiogenesis inhibitor-free NACT regimens in all included randomized controlled trials (the size of the squares is proportional to the weight of each study). **(B)** Funnel plot with pseudo 95% confidence limits for the effect of angiogenesis inhibitor-based NACT estimated from individual studies (horizontal axis) against the study size (vertical axis): the symmetric inverted funnel shape suggests a low likelihood of publication bias.

Overall, among all 9 randomized controlled trials, 1,437 out of 5,478 patients (26.23%) achieved pCR following neoadjuvant therapy. Specifically, pCR was attained by 818 out of 2,738 patients (29.88%) in the angiogenesis inhibitor chemotherapy group and 619 out of 2,740 patients (22.59%) in the chemotherapy-alone group (OR 1.49, 95% CI 1.31–1.68, P = 0.56; I^2^ = 0%, P < 0.0001) (**Figure 2A**). In addition, funnel plot analysis indicated no evidence of publication bias (**Figure 2B**). Detailed sensitivity analyses of these results are provided in **Supplementary Table 1**, accessible online in Annals of Oncology.

**Figure 3:**
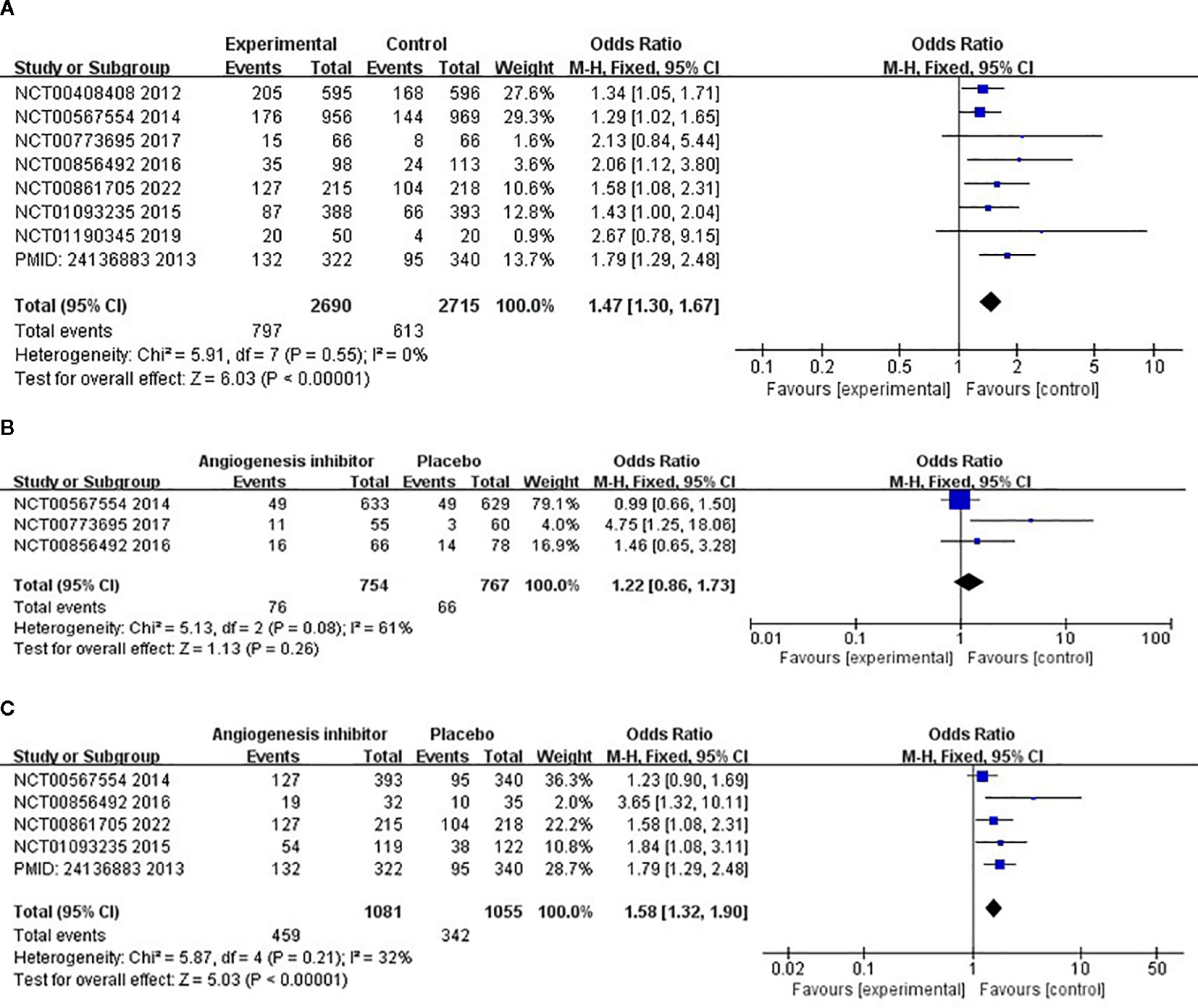
Odds ratios for pathological complete response with angiogenesis inhibitor-based versus angiogenesis inhibitor-free NACT in randomized controlled trials, stratified as follows: **(A)** Both treatment arms received anthracycline- and taxane-based chemotherapy regimens. **(B)** Both treatment arms comprised HR(+) breast cancer patients. **(C)** Both treatment arms comprised TNBC patients. The size of the squares is proportional to the weight of each study.

Among the 8 randomized controlled trials utilizing anthracycline and taxane-based chemotherapy, 1,410 out of 5,405 patients (26.09%) achieved pCR. This includes 797 out of 2,690 patients (29.63%) in the angiogenesis inhibitor group and 613 out of 2,715 patients (22.58%) in the control group (OR 1.47, 95% CI 1.30–1.67, P = 0.55; I^2^ = 0%, P < 0.0001) (**Figure 3**). Detailed sensitivity analyses of these results are provided in **Supplementary Table 2**, accessible online in Annals of Oncology.

In three RCTs focusing on HR(+) patients (21–23), 142 out of 1,521 patients (9.34%) achieved pCR. This included 76 out of 754 patients (10.08%) in the angiogenesis inhibitor group and 66 out of 767 patients (8.60%) in the control group (OR 1.22, 95%CI0.86-1.73,P=0.08;I2 = 61%,P=0.26) (**Figure 3B**).

In five randomized controlled trials involving TNBC patients (21, 23–25, 28), 801 out of 2,136 patients (37.5%) achieved pCR. This included 459 out of 1,081 patients (42.46%) in the angiogenesis inhibitor group and 342 out of 1,055 patients (32.42%) in the control group (OR 1.58, 95% CI 1.32–1.90, P = 0.21; I^2^ = 32%, P < 0.00001) (**Figure 3C**).

### Objective response rate

**Figure 4:**
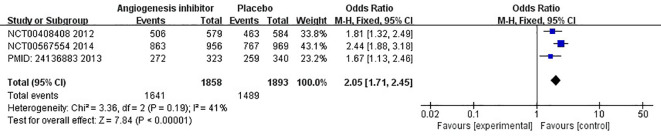
Method of assessment of objective response rate in the included RCTs.

Three randomized controlled trials reported the ORR at the end of NACT (19, 21). Overall, the ORR after NACT was 83.44% (3,130 out of 3,751 patients). Specifically, the ORR was 88.32% (1,641 out of 1,858 patients) in the angiogenesis inhibitor chemotherapy group, compared to 78.66% (1,489 out of 1,893 patients) in the chemotherapy-alone group. The OR for these findings was 2.05, with a 95% confidence interval of 1.71 to 2.45 (P = 0.19; I^2^ = 41%, P < 0.00001) (**Figure 4**). Sensitivity analyses are provided in **Supplementary Table 3**, available online in Annals of Oncology.

### OS, DFS

Several RCTs reported survival outcomes. Some trials reported only OS, while others focused solely on DFS. Specifically, seven RCTs documented OS results (20, 21, 23–27). The median follow-up periods were as follows: 56 months in the NCT00528567 trial, 45.6 months in the NCT00567554 trial, 36 months in the NCT00856492 trial, 94.8 months in the NCT00861705 trial, 42 months in the NCT01093235 trial, 62.4 months in the NCT01142778 trial, and 60.9 months in the NCT01190345 trial. Four RCTs reported DFS data (21, 25–27). Additionally, two RCTs reported event-free survival (EFS) outcomes.

**Figure 5:**
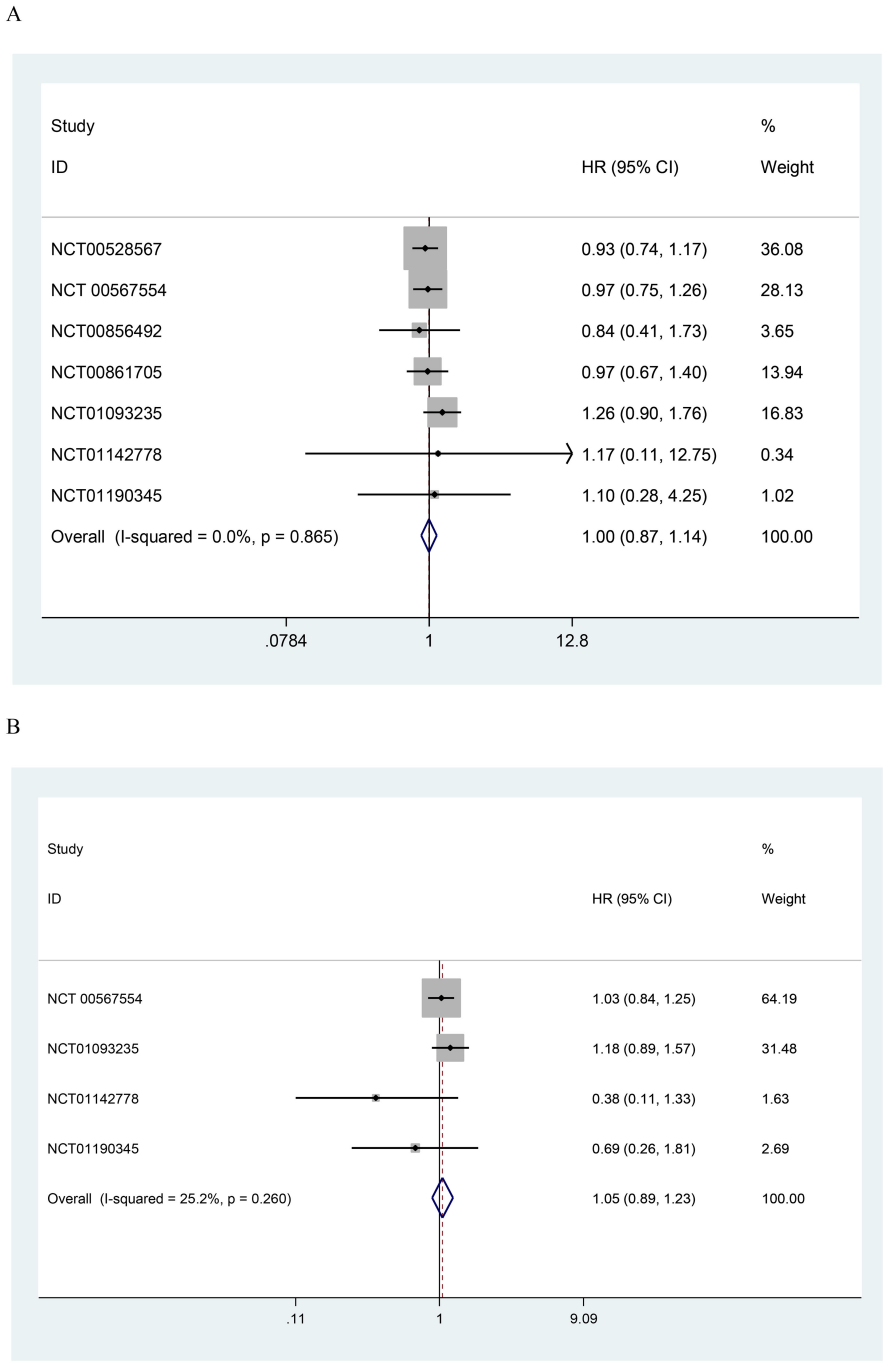
Hazard ratios for OS **(A)** and distant DFS **(B)** of angiogenesis inhibitor-based versus angiogenesis inhibitor-free neoadjuvant chemotherapy (NACT). The size of the squares is proportional to the weight of each study.

In summary, angiogenesis inhibitors did not improve OS (HR 1.00, 95% CI 0.87–1.14, P = 0.957; I^2^ = 0.0%, P = 0.865) (**Figure 5A**), nor DFS (HR 1.05, 95% CI 0.89–1.23, P = 0.579; I^2^ = 25.2%, P = 0.260) (**Figure 5B**).

### Adverse events

**Figure 6:**
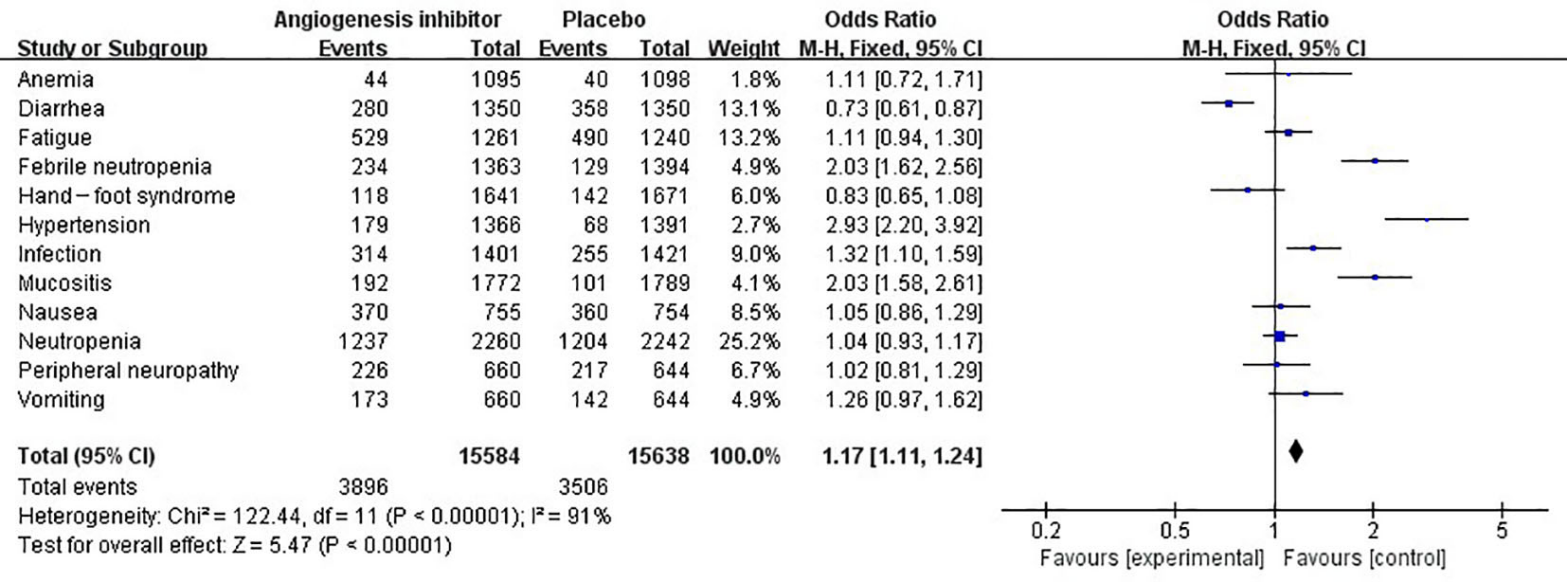
Overview of safety profile. Odds ratios for adverse reactions in angiogenesis inhibitor-based versus angiogenesis inhibitor-free NACT.

In this study, a total of 12 AEs were collected; however, it is important to note that these events did not necessarily occur simultaneously across all trials. The OR was 1.17 (95% confidence interval 1.11–1.24, P < 0.00001; I^2^ = 91%, P < 0.00001). **Figure 6** presents the safety profile of AEs, comparing the angiogenesis inhibitor-containing chemotherapy group and the chemotherapy-alone group.

### Neutropenia

**Figure 7:**
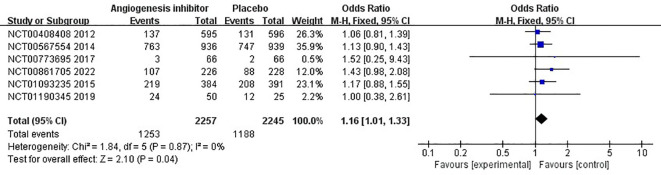
Odds ratios for neutropenia in angiogenesis inhibitor-based versus angiogenesis inhibitor-free NACT.

Six randomized controlled trials reported the incidence of neutropenia. Overall, 2,441 out of 4,502 patients (54.22%) developed neutropenia after neoadjuvant therapy. Specifically, in the chemotherapy group with angiogenesis inhibitors, 1,253 out of 2,257 patients (55.52%) experienced neutropenia, while in the chemotherapy group without angiogenesis inhibitors, only 1,188 out of 2,245 patients (52.92%) developed neutropenia (OR 1.16, 95% CI 1.01–1.33, P < 0.04; I^2^ = 0%, P = 0.87) (**Figure 7**). Detailed sensitivity analyses of these results are provided in **Supplementary Table 4**, accessible online in Annals of Oncology.

### Hypertension

**Figure 8:**
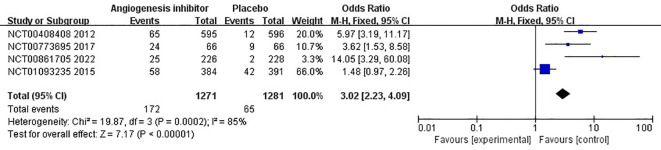
Odds ratios for hypertension in angiogenesis inhibitor-based versus angiogenesis inhibitor-free NACT.

Four randomized controlled trials reported the incidence of hypertension. Overall, 237 of 2552 patients (9.29%) developed hypertension following neoadjuvant therapy. Specifically, hypertension occurred in 172 of 1271 patients (13.53%) in the angiogenesis inhibitor chemotherapy group, compared with 65 of 1281 patients (5.07%) in the chemotherapy-alone group (OR 3.02, 95% CI 2.23–4.09, P < 0.00001; I^2^ = 85%, P = 0.0002) (**Figure 8**). Detailed sensitivity analyses of these results are provided in **Supplementary Table 5**, accessible online in Annals of Oncology.

### Febrile neutropenia

**Figure 9:**
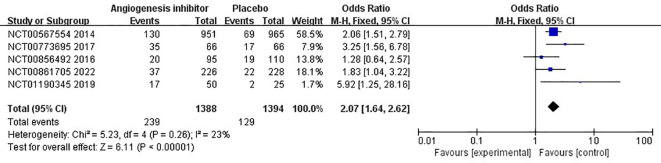
Odds ratios for febrile neutropenia in angiogenesis inhibitor-based versus angiogenesis inhibitor-free NACT.

Five randomized controlled trials reported the incidence of febrile neutropenia. Overall, 368 out of 2782 patients (13.23%) developed febrile neutropenia after neoadjuvant therapy. Specifically, in the chemotherapy group with angiogenesis inhibitors, 239 out of 1388 patients (17.22%) experienced febrile neutropenia, whereas in the chemotherapy group without angiogenesis inhibitors, only 129 out of 1394 patients (9.25%) developed febrile neutropenia (OR 2.07, 95% CI 1.64–2.62, P < 0.00001; I^2^ = 23%, P = 0.26) (**Figure 9**). Detailed sensitivity analyses of these results are provided in **Supplementary Table 6**, accessible online in Annals of Oncology.

### Mucositis

Three randomized controlled trials reported the incidence of mucositis. Overall, 293 out of 3561 patients (8.23%) developed mucositis after neoadjuvant therapy. Specifically, in the chemotherapy group with angiogenesis inhibitors, 192 out of 1772 patients (10.84%) experienced mucositis, whereas in the chemotherapy group without angiogenesis inhibitors, only 101 out of 1789 patients (5.65%) developed mucositis.

### Nausea

**Figure 10:**
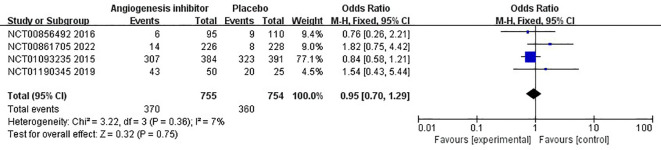
Odds ratios for febrile nausea in angiogenesis inhibitor-based versus angiogenesis inhibitor-free NACT.

Four randomized controlled trials reported the incidence of nausea. Overall, 730 out of 1509 patients (48.38%) developed nausea after neoadjuvant therapy. Specifically, in the chemotherapy group with angiogenesis inhibitors, 370 out of 755 patients (49.01%) experienced nausea, whereas in the chemotherapy group without angiogenesis inhibitors, 360 out of 754 patients (47.75%) developed nausea (OR 0.95, 95% CI 0.70–1.29, P = 0.75; I^2^ = 7%, P = 0.36) (**Figure 10**). Detailed sensitivity analyses of these results are provided in **Supplementary Table 7**, accessible online in Annals of Oncology.

## Discussion

This meta-analysis comprehensively summarizes evidence from RCTs evaluating the activity, efficacy, and safety of anti-angiogenic agents as part of NACT for breast cancer patients. Our findings indicate that while the addition of anti-angiogenic agents to NACT significantly improves pCR rates and ORR, it comes at the cost of increased hematological toxicity. No significant differences were observed in OS or DFS.

Bevacizumab effectively blocks tumor angiogenesis by inhibiting the vascular endothelial growth factor (VEGF) signaling pathway, thereby reducing tumor blood supply and nutritional support. This anti-angiogenic effect not only enhances the intratumoral delivery efficiency of chemotherapy drugs by promoting vascular normalization but also improves the tumor microenvironment. Studies have shown that while reducing vascular density (particularly in angiopoietin-2-positive vessels), the drug promotes the infiltration of CD4+ T cells, CD8+ T cells, and mature dendritic cells into tumors. This immunomodulatory effect provides a theoretical basis for its combination with immune checkpoint inhibitors, particularly in the treatment of TNBC (29). When bevacizumab is combined with NACT, its efficacy-enhancing mechanisms may include: optimizing the distribution of chemotherapy drugs within tumors through vascular normalization (6), and suppressing the pro-angiogenic activity of tumor-associated macrophages—although the specific pathways involved still require comprehensive elucidation (30). It is noteworthy that long-term use may lead to excessive vascular depletion, resulting in a hypoxic microenvironment that could potentially induce chemotherapy resistance or tumor recurrence (6).

Bevacizumab induces hypertension by inhibiting the VEGF signaling pathway, leading to vasoconstriction and endothelial dysfunction (31). VEGF normally binds to VEGFR2 on endothelial cells and helps make nitric oxide (NO) to widen blood vessels. Bevacizumab takes away VEGF so less NO is made. This causes blood vessels to tighten and blood pressure to go up. The chemotherapy type can also change the risk. Anthracycline drugs (like epirubicin/cyclophosphamide) damage blood vessels and the heart and can make the blood pressure rise worse with bevacizumab. Taxane drugs (like docetaxel) cause fluid buildup and may change how the body handles VEGF blockade. These drug effects likely explain the high heterogeneity (I^2^ = 85%).Consequently, hypertension is recognized as one of the most common AEs associated with this drug. For instance, in bevacizumab monotherapy, hypertension (8.3%) ranks as the second most frequent grade ≥3 adverse event (32), while in combination therapies such as with sintilimab, the incidence of hypertension remains notably high (11.8%) (33). Additionally, patients with pre-existing hypertension or diabetes are more susceptible to treatment interruption due to bevacizumab (34). We saw high heterogeneity (I^2^ = 85%) in the four RCTs on hypertension. The pooled OR (3.02, 95% CI 2.23–4.09) is a general trend not a precise number. The heterogeneity came from different hypertension grading rules (different CTCAE versions), different blood pressure check methods, different baseline risk factors (like high blood pressure, diabetes, and obesity), and unreported other drugs (like blood pressure pills and steroids). This pooled estimate only shows that bevacizumab raises the risk of hypertension.

The differing statistical outcomes between neutropenia and febrile neutropenia can be explained as follows: Neutropenia, a common chemotherapy-related adverse effect, may exhibit similar incidence rates across different treatment regimens due to baseline risks (e.g., chemotherapy intensity) or prophylactic measures (e.g., granulocyte colony-stimulating factor [G-CSF] administration), resulting in non-significant intergroup differences (35). In contrast, febrile neutropenia depends not only on the degree of neutropenia but also on infection-triggering factors. Bevacizumab may impair mucosal barrier repair by inhibiting VEGF, thereby increasing the risk of infections and predisposing patients to febrile neutropenia. Furthermore, the incidence of febrile neutropenia is higher (12%) among elderly female patients receiving bevacizumab-containing regimens. Although febrile neutropenia is generally considered a low-risk event, the heightened susceptibility to infections in this population may amplify the statistical differences observed (36).

In subgroup analyses, the pCR rate was significantly higher in patients with TNBC than in those with HR(+) breast cancer, and this difference was statistically significant. The underlying reasons primarily stem from biological differences between the two breast cancer subtypes. First, TNBC exhibits higher expression of VEGF and greater angiogenic activity (30). As an anti-VEGF agent, bevacizumab may significantly enhance TNBC’s sensitivity to chemotherapy by inhibiting tumor angiogenesis. In contrast, HR(+) breast cancer is less dependent on angiogenesis, which limits the additional benefit conferred by bevacizumab (37). Second, the tumor microenvironment in TNBC shows more substantial immune cell infiltration, such as tumor-associated macrophages, which can influence treatment response by mediating immunosuppression or promoting angiogenesis. Bevacizumab may further enhance chemotherapy efficacy by modulating the immune microenvironment, for instance, by reducing immunosuppressive cells (38). However, the lower degree of immune infiltration in HR(+) breast cancer restricts this synergistic effect (39). In summary, the active angiogenic features and distinct immune microenvironment of TNBC collectively contribute to a more pronounced improvement in pCR rates with bevacizumab, whereas HR(+) breast cancer derives limited benefit due to its differing biological characteristics. We saw moderate heterogeneity (I^2^ = 61%) in the three RCTs on HR+ breast cancer patients. This came from different HR+ cutoff values (like ≥1% vs. ≥10%), different chemotherapy regimens (like taxane drugs and anthracycline doses), no standard way to use endocrine therapy, and different patient baseline features (like menopausal status and Ki-67 index). So we need to be careful with the pooled result for HR+ patients (OR 1.22, 95% CI 0.86–1.73). The lack of statistical significance may come from both limited real efficacy and clinical heterogeneity across studies.

Adding angiogenesis inhibitors to neoadjuvant chemotherapy improves pCR rates. This improvement did not lead to better OS or DFS in our study. Why does better pCR not give a survival benefit? We have a few possible reasons. First, pCR measures local tumor response in the breast and lymph nodes. OS and DFS depend more on distant metastasis and post-surgery treatments. A better pCR does not always mean fewer distant relapses. Second, breast cancer cells quickly become resistant to anti-angiogenic drugs like bevacizumab. Short-term VEGF inhibition can make tumors more sensitive to chemotherapy and raise pCR. But resistance develops fast. This leads to later treatment failure and cancels out the early survival benefit from a good pCR. Third, chemotherapy has known toxic effects. Adding angiogenesis inhibitors does not lower these toxicities and may add more side effects like hypertension. Long-term toxicity may weaken any small survival benefit from a higher pCR. Fourth, many studies show a weak link between pCR and long-term survival, especially in some subgroups. Some patients still relapse from micrometastases or tumor heterogeneity after they achieve pCR. Also, current anti-angiogenic drugs may not fully block tumor blood vessel growth. They have poor tumor penetration and limited efficacy. So their effect on long-term survival is small.

Bevacizumab improves pCR rates with neoadjuvant chemotherapy, especially in TNBC. But it does not improve overall survival, and treatment response varies a lot. This shows we need predictive biomarkers. Right now, no validated biomarker tells us which breast cancer patients will get the most benefit from adding angiogenesis inhibitors to neoadjuvant therapy.

We have some candidate biomarkers for future study. Tumor VEGF-A expression levels might predict response, but study results are not consistent. This is partly because of differences in testing methods and tumor heterogeneity. Circulating angiogenic factors like plasma VEGF, soluble VEGFR2, and angiopoietin-2 might show real-time changes in tumor blood vessels during treatment (40).They could offer a non-invasive way to monitor treatment. Tumor-infiltrating lymphocytes (TILs) and immune-related gene signatures have also gained attention. Bevacizumab can change the tumor immune environment by increasing CD8+ T cells and lowering immunosuppressive cells like M2 macrophages (41). Genomic signatures linked to angiogenesis pathways, like a 10-gene hypoxia signature or a 19-gene angiogenic signature, have shown early promise in retrospective analyses of the GeparQuinto and BEATRICE trials. But they need prospective validation (42, 43).

New high-throughput technologies like single-cell RNA sequencing and spatial transcriptomics help us study how angiogenesis inhibition, immune modulation, and chemotherapy sensitivity work together. Liquid biopsy methods like circulating tumor DNA (ctDNA) and circulating tumor cell (CTC) analysis can also track resistance mutations during treatment (44). These mutations might be in VEGFR2 or in downstream pathways like RAS/MAPK and PI3K/AKT (45).

Future prospective clinical trials should plan ahead for biomarker collection and analysis. They should move away from a one-size-fits-all approach. We need to combine multi-omics profiling with long-term biospecimen collection. This will help us build a strong set of biomarkers to guide patient selection and balance risks and benefits.

## Conclusion

Bevacizumab combined with NACT can increase the pCR rate, particularly in TNBC. However, these studies have not consistently demonstrated an OS benefit, and there is a lack of clear predictive biomarkers.

## Data Availability

The original contributions presented in the study are included in the article/**Supplementary Material**. Further inquiries can be directed to the corresponding author.
