# Supplementary material for: Neoadjuvant chemotherapy with angiogenesis inhibitor in early-stage breast cancer: a systematic review and meta-analysis
Source: Front Oncol. 2026 Jul 15;16:1830676. doi: 10.3389/fonc.2026.1830676 (PMC13414213; doi:10.3389/fonc.2026.1830676)
Supplement: Supplementary file 1 [file Table1.docx]

**Supplementary Table S1.** Sensitivity analysis performed excluding each study at a time to assess the stability of the estimated pooled odds ratio for pathological complete response in the included randomized controlled trials

| Study excluded | Random effect | | I-squared (%) | I-sq. P-value |
| --- | --- | --- | --- | --- |
|  | OR | 95% CI |  |  |
| NCT00408408 | 1.54 | 1.33-1.78 | 0% | 0.56 |
| NCT 00567554 | 1.56 | 1.35-1.81 | 0% | 0.65 |
| NCT00773695 | 1.48 | 1.30-1.67 | 0% | 0.52 |
| NCT00856492 | 1.46 | 1.29-1.66 | 0% | 0.59 |
| [NCT00861705](https://www.clinicaltrials.gov/ct2/show/NCT00861705) | 1.47 | 1.29-1.68 | 0% | 0.47 |
| NCT01093235 | 1.49 | 1.31-1.71 | 0% | 0.46 |
| NCT01142778 | 1.47 | 1.30-1.67 | 0% | 0.55 |
| NCT01190345 | 1.48 | 1.30-1.67 | 0% | 0.55 |
| PMID: 24136883 | 1.44 | 1.26-1.65 | 0% | 0.63 |

**Supplementary Table S2.** Sensitivity analysis performed excluding each study at a time to assess the stability of the estimated pooled odds ratio for pathological complete response in the included randomized controlled trials

| Study excluded | Random effect | | I-squared (%) | I-sq. P-value |
| --- | --- | --- | --- | --- |
|  | OR | 95% CI |  |  |
| NCT00408408 | 1.53 | 1.32-1.77 | 0% | 0.53 |
| NCT 00567554 | 1.55 | 1.34-1.80 | 0% | 0.63 |
| NCT00773695 | 1.46 | 1.29-1.66 | 0% | 0.51 |
| NCT00856492 | 1.45 | 1.28-1.65 | 0% | 0.58 |
| [NCT00861705](https://www.clinicaltrials.gov/ct2/show/NCT00861705) | 1.46 | 1.28-1.67 | 0% | 0.45 |
| NCT01093235 | 1.48 | 1.29-1.70 | 0% | 0.44 |
| NCT01190345 | 1.46 | 1.29-1.66 | 0% | 0.54 |
| PMID: 24136883 | 1.42 | 1.24-1.63 | 0% | 0.64 |

**Supplementary Table S3.** Sensitivity analysis performed excluding each study at a time to assess the stability of the estimated pooled odds ratio for objective response rate in the included randomized controlled trials.

| Study excluded | Random effect | | I-squared (%) | I-sq. P-value |
| --- | --- | --- | --- | --- |
|  | OR | 95% CI |  |  |
| NCT00408408 | 2.17 | 1.75-2.70 | 60% | 0.11 |
| NCT 00567554 | 1.75 | 1.37-2.24 | 0% | 0.75 |
| PMID: 24136883 | 2.17 | 1.77-2.65 | 51% | 0.15 |

**Supplementary Table S4.** Sensitivity analysis performed excluding each study at a time to

assess the stability of the estimated pooled odds ratio for neutropenia in the included randomized controlled trials.

| Study excluded | Random effect | | I-squared (%) | I-sq. P-value |
| --- | --- | --- | --- | --- |
|  | OR | 95% CI |  |  |
| NCT00408408 | 1.19 | 1.02-1.40 | 0% | 0.86 |
| NCT 00567554 | 1.17 | 0.99-1.39 | 0% | 0.77 |
| NCT00773695 | 1.16 | 1.01-1.33 | 0% | 0.78 |
| [NCT00861705](https://www.clinicaltrials.gov/ct2/show/NCT00861705) | 1.12 | 0.97-1.30 | 0% | 0.98 |
| NCT01093235 | 1.15 | 0.99-1.35 | 0% | 0.77 |
| NCT01190345 | 1.16 | 1.01-1.33 | 0% | 0.78 |

**Supplementary Table S5.** Sensitivity analysis performed excluding each study at a time to

assess the stability of the estimated pooled odds ratio for hypertension in the included randomized controlled trials.

| Study excluded | Random effect | | I-squared (%) | I-sq. P-value |
| --- | --- | --- | --- | --- |
|  | OR | 95% CI |  |  |
| NCT00408408 | 2.28 | 1.61-3.25 | 82% | 0.004 |
| NCT00773695 | 2.95 | 2.14-4.07 | 90% | 0.001 |
| [NCT00861705](https://www.clinicaltrials.gov/ct2/show/NCT00861705) | 2.64 | 1.93-3.62 | 86% | 0.008 |
| NCT01093235 | 6.02 | 3.75-9.66 | 24% | 0.27 |

**Supplementary Table S6.** Sensitivity analysis performed excluding each study at a time to

assess the stability of the estimated pooled odds ratio for febrile neutropenia in the included randomized controlled trials.

| Study excluded | Random effect | | I-squared (%) | I-sq. P-value |
| --- | --- | --- | --- | --- |
|  | OR | 95% CI |  |  |
| NCT 00567554 | 2.09 | 1.46-3.00 | 43% | 0.16 |
| NCT00773695 | 1.97 | 1.54-2.52 | 15% | 0.32 |
| NCT00856492 | 2.20 | 1.71-2.82 | 7% | 0.36 |
| [NCT00861705](https://www.clinicaltrials.gov/ct2/show/NCT00861705) | 2.12 | 1.64-2.74 | 41% | 0.17 |
| NCT01190345 | 2.00 | 1.58-2.54 | 12% | 0.33 |

**Supplementary Table S7.** Sensitivity analysis performed excluding each study at a time to

assess the stability of the estimated pooled odds ratio for febrile nausea in the included randomized controlled trials.

| Study excluded | Random effect | | I-squared (%) | I-sq. P-value |
| --- | --- | --- | --- | --- |
|  | OR | 95% CI |  |  |
| NCT00856492 | 0.97 | 0.70-1.34 | 34% | 0.22 |
| [NCT00861705](https://www.clinicaltrials.gov/ct2/show/NCT00861705) | 0.87 | 0.62-1.20 | 0% | 0.64 |
| NCT01093235 | 1.33 | 0.73-2.40 | 0% | 0.45 |
| NCT01190345 | 0.92 | 0.67-1.27 | 24% | 0.27 |
